# Electrosynthesis of Hydrogen Peroxide at Industrial‐Level Current Density in Flow‐Cell System: Interfacial Microenvironment Regulation and Catalyst Design

**DOI:** 10.1002/smsc.202500017

**Published:** 2025-05-19

**Authors:** Abdalazeez Ismail Mohamed Albashir, Yunlong Li, Jing Dou, Ke Qi, Wei Qi

**Affiliations:** ^1^ School of Materials Science and Engineering University of Science and Technology of China Shenyang Liaoning 110016 P. R. China; ^2^ Shenyang National Laboratory for Materials Science Institute of Metal Research Chinese Academy of Sciences Shenyang Liaoning P. R. China

**Keywords:** flow‐cell, hydrogen peroxide, interfacial microenvironment, mesoporous structures, two‐electron oxygen reduction

## Abstract

Electrosynthesis of hydrogen peroxide via two‐electron oxygen reduction (2e^−^ ORR) provides a green, sustainable, and cost‐effective alternative to anthraquinone processes. However, scaling up from laboratory evaluations to practical applications remains challenging. Herein, an interfacial microenvironment regulation strategy using cetyltrimethylammonium bromide cationic surfactant is reported to boost the hydrogen peroxide (H_2_O_2_) production rate of commercial carbon black catalysts in alkaline flow‐cell reactors. The modified interfacial microenvironment creates an ideal environment for H_2_O_2_ production, resulting in a 1.40‐fold improvement in 2e^−^ ORR current density (from 227.0 to 320.0 mA cm^−2^) and a 1.58‐fold improvement in H_2_O_2_ production rate (from 137.0 to 217.8 mM L^−1^ h^−1^). Additionally, a boron‐doped mesoporous carbon catalyst is developed, demonstrating superior catalytic performance, achieving a 1.80‐fold improvement in H_2_O_2_ production rate (246.7 mM L^−1^ h^−1^) comparing with commercial carbon black. These results highlight the potential of microenvironment regulation and catalyst design for developing highly efficient and scalable H_2_O_2_ electrosynthesis system.

## Graphical abstract

The present study highlights the underlying mechanism behind cationic surfactant‐modified interfacial microenvironment engineering strategy for enhanced two‐electron oxygen reduction process. The kinetic analysis demonstrates that the strengthened electric field stabilizes HO_2_^−^ intermediates, promoting efficient H_2_O_2_ production. Furthermore, a novel boron‐doped mesoporous carbon with a unique tailored porous structure is developed, achieving industrial‐level H_2_O_2_ production with 100% Faradic efficiency.

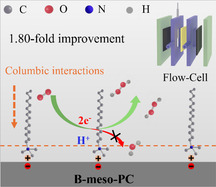

## 1 Introduction

The electrochemical reduction of earth‐abundant oxygen molecules (O_2_) into high value‐added hydrogen peroxide (H_2_O_2_) via the two‐electron oxygen reduction reaction (2e^−^ ORR) represents a promising and sustainable alternative to the waste‐intensive conventional anthraquinone oxidation process.^[^ ^1^ ^]^ The state‐of‐the‐art electrocatalytic materials, including metal‐based material and their alloys such as Pd,^[^ ^2^ ^]^ Pt,^[^ ^3^ ^]^ Co–N,^[^ ^4^ ^]^ and Ni–N,^[^ ^5^ ^]^ as well as metal‐free carbon‐based materials,^[^ ^6^, ^7^, ^8^, ^9^, ^10^, ^11^ ^]^ have shown significant potential as electrocatalysts for 2e^−^ ORR process. Although the development of active catalysts has been the central focus of the 2e^−^ ORR process in recent years,^[^ ^12^, ^13^, ^14^ ^]^ the overall selectivity and efficiency of the reaction system are significantly influenced by the characteristics of the interfacial microenvironment at the catalyst–electrolyte–gas triple‐phase boundary, where the availability and arrangement of reactants, intermediates, and active sites play a vital roles.^[^ ^15^, ^16^, ^17^, ^18^ ^]^ The selectivity and yield of the target product H_2_O_2_ are impeded by competing side reactions, such as the four‐electron oxygen reduction reaction (4e^−^ ORR) to H_2_O and the hydrogen evolution reaction (HER).^[^ ^19^ ^]^ As a result, the efficient diffusion/transportation of oxygen, protons, electrons, and other reaction intermediates within the interfacial microenvironment plays a crucial role for the highly efficient H_2_O_2_ production.

Recent studies highlighted the effectiveness of interfacial microenvironment engineering strategies in advancing the 2e^−^ ORR process, providing crucial in‐depth physicochemical insights into on‐site H_2_O_2_ electrochemical production.^[^ ^20^, ^21^, ^22^ ^]^ For example, it has been demonstrated that the addition of polar organic solvents can regulate the activity of the water hydrogen‐bond network at the electrode interface by minimizing the water dissociation into hydroxide ions (OH^−^) and protons (H^+^), consequently preventing the 4e^−^ ORR and HER process and providing an optimal proton transfer supply favoring the 2e^−^ ORR over competition reactions, achieving over 90.0% H_2_O_2_ selectivity.^[^ ^23^ ^]^ Metal cations (Na, K, Cr, Li) have also been applied to effectively hinder the accessibility of the interfacial hydrogen‐bond network, thereby lowering the hydrogen transfer rate and consequently inhibiting HER process.^[^ ^24^ ^]^ In addition, the incorporation of cationic surfactants into the electrolyte solution has been found to play a crucial role in regulating the interfacial microenvironment, facilitating the 2e^−^ ORR process on carbon materials.^[^ ^21^ ^]^ Despite extensive research, significant gap remains in the field. Most present studies focused on conventional H‐cells or rotating ring‐disk electrode (RRDE) systems,^[^ ^25^ ^]^ which lack insights into practical application. These systems also fail to achieve the high current densities required for industrial‐scale H_2_O_2_ production. Consequently, in‐depth investigations and understandings are mandatory to assess the impacts of these microenvironmental modifications in advanced configurations, such as flow‐cell reactors. Flow‐cell reactors can not only meet the on‐site production demands but also capable of delivering high current density required for potential industrial‐scale applications.^[^ ^26^, ^27^, ^28^, ^29^ ^]^

**Scheme 1:**
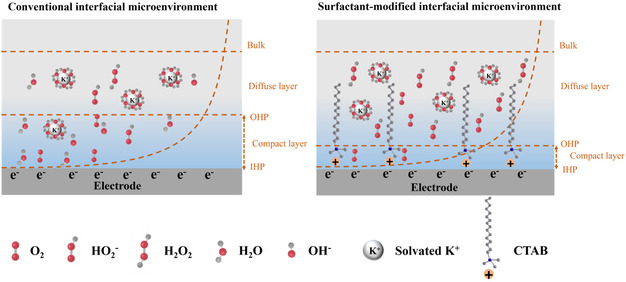
Schematic illustration of surfactant‐modified interfacial microenvironment.

In addition, the physicochemical nature behind the effect of these electrolyte additives, such as cetyltrimethylammonium bromide (CTAB) surfactants in these environments and especially their underlying mechanism for promoting H_2_O_2_ production rate and reaction kinetics, remains underexplored. Bridging this gap is crucial for developing efficient and scalable electrochemical system for H_2_O_2_ production. Aiming at these scientific challenges, we have reported a cationic surfactant‐modified interfacial microenvironment strategy (**Scheme** **1**) in the present study to boost the production rate of H_2_O_2_ for 2e^−^ ORR in an alkaline flow‐cell reactor. The introduction of CTAB into the electrolyte solution results in a substantial increase in 2e^−^ ORR current density from 227.0 to 320.0 mA cm^−2^ and corresponding H_2_O_2_ production rate from 137.0 to 217.8 mM L^−1^ h^−1^ representing a 1.58‐fold improvement. The enhanced H_2_O_2_ production rate is found to be attributed to the accumulated charge and strengthened local electric field at the compact layer. This phenomenon arises from selective adsorption of the positively charged head group of CTAB surfactant molecules onto the electrode surface via Coulombic force. As a result, the adsorption optimizes interfacial conductivity by facilitating charge transfer and the strengthening electric double layer, thereby accelerating reaction kinetics. In addition to surfactant modification, the carbon catalyst is also optimized via heteroatom doping and porous engineering strategies.^[^ ^30^ ^]^ A novel boron‐doped mesoporous carbon (B‐meso‐PC) has demonstrated an improved catalytic activity, achieving a remarkable H_2_O_2_ production rate of 246.7 mM L^−1^ h^−1^ and higher industrial‐relevant current density of 382.0 mA cm^−2^, comparing with conventional carbon black (CB) catalysts. This enhancement should be attributed to the synergistic effect of the well‐designed interfacial microenvironment, unique tailored porous structure, and boron heteroatom doping. The present work not only provides a novel nonmetallic reaction system within flow‐cell configuration for highly efficient electrochemical synthesis of H_2_O_2_ but also emphasizes the importance of in‐depth understandings on the nature of the promotion effect, shedding light on the potential practical applications of onsite electrochemical synthesis of H_2_O_2_ via 2e^−^ ORR process.

## 2 Results and Discussion

### 2.1 2e^−^ ORR Catalytic Activity of Carbon in a Cationic Surfactant‐Modified Interfacial Microenvironment

**Figure 1:**
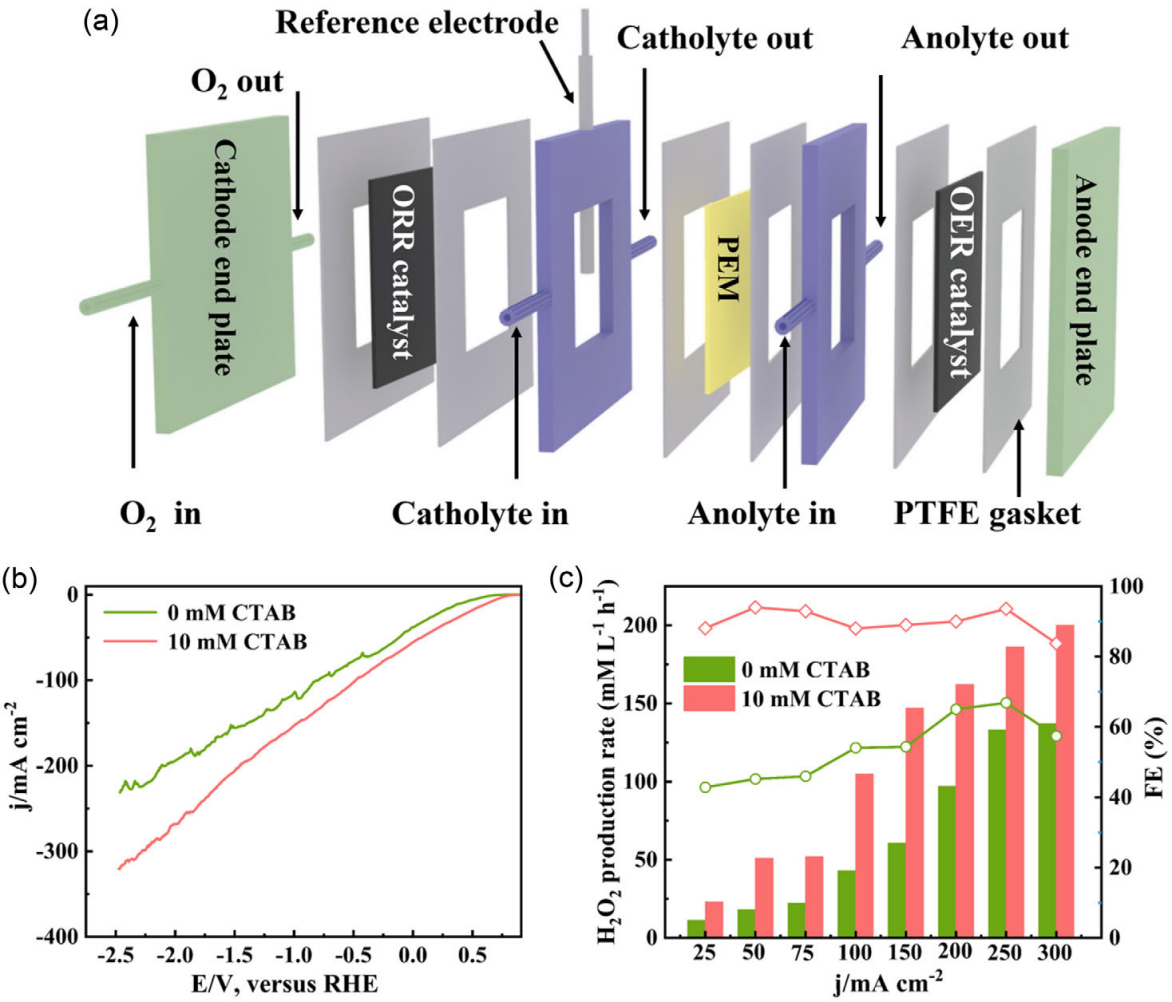
H_2_O_2_ production performance in flow‐cell reactor. a) Schematic illustration of the flow‐cell reactor setup for H_2_O_2_ production, b) LSV curve, and c) H_2_O_2_ production rate and corresponding FE (%) of CB in the absence and in the presence of CTAB (10 mM).

A three‐electrode flow‐cell reactor (**Figure** **1**a) and commercial CB (Figure S2a,b, Supporting Information) was selected to study the impact of a cationic surfactant‐modified interfacial microenvironment on H_2_O_2_ production rate and reaction kinetics. The flow‐cell reactor reaction setup offers a robust platform to study the roles of CTAB cationic surfactants under practical operational conditions. The electrolyte solution of 1.0 M KOH (0 mM CTAB) and 1.0 M KOH‐containing CTAB (2.0–20.0 mM) were recycled at a constant flow rate of 5.0 mL min^−1^. As shown in Figure 1b, the linear sweep voltammetry (LSV) results indicate that the CB catalyst electrode in the presence of CTAB exhibited much higher 2e^−^ ORR current density of 320.0 mA cm^−2^ than that without CTAB of 227.0 mA cm^−2^, representing a 1.40‐flod improvement for the electrochemical synthesis of H_2_O_2_. The H_2_O_2_ production rate and Faradic efficiency (FE) at various applied current densities (25.0 to 300.0 mA cm^−2^) are displayed in Figure 1c, showing that the H_2_O_2_ production rate reached 200 mM L^−1^ h^−1^ with FE of 83.8% at a current density of 300.0 mA cm^−2^ in 10 mM CTAB, which is much higher than that in the absence of CTAB (137.0 mM L^−1^ h^−1^ with FE of 57.3%). Even at lower current densities, the H_2_O_2_ production rate and FE% remained higher, achieving 23.0 mM L^−1^ h^‐1^ with FE of 88.0% at 25.0 mA cm^−2^ and 51.0 mM L^−1^ h^−1^ with FE of 94.0% at 50.0 mA cm^−2^, respectively, highlighting the significant role of the CTAB surfactant in promoting the 2e^−^ ORR activity. It is interesting to observe from the high‐resolution N 1*s* X‐ray photoelectron spectroscopy (XPS) spectra of the electrode after electrochemical reactions (Figure S3, Supporting Information) that there is an obvious nitrogen signal, indicating that some of CTAB molecules are firmly adsorbed on the electrode surface Notably, the strong signal from nitrogen (N) and the trace amounts of bromine (Br), with no additional decomposition products observed in the XPS spectra (Table S1, Supporting Information), suggest the selective adsorption of the positively charged ammonium head group of CTAB on electrode surface and remains stable without significant degradation during electrochemical operation. This confirms that CTAB consistently modifies the interfacial microenvironment and enhances H_2_O_2_ selectivity under the applied conditions. However, in light of the environmental issues related to surfactant removal, future studies might investigate biodegradable alternatives,^[^ ^31^ ^]^ which provide comparable interfacial modifications while reducing environmental hazards. It should be noted that the H_2_O_2_ production rate and FE (%) would significantly decrease to 37.0 mM L^−1^ h^−1^ and 31.0%, respectively, in the presence of the anionic surfactant (sodium 1‐hexadecylsulfonate,10 mM), as shown in Figure S4, Supporting Information. This decrease should be attributed to the formed diffusion barrier at the outer Helmholtz plane, caused by the negatively charged head group of anionic surfactants, which impedes the efficient transportation of reactants and HO_2_^−^ intermediates from electrode surface.^[^ ^32^ ^]^ These results suggest that the cationic surfactant‐modified interfacial microenvironment is a promising strategy to improve H_2_O_2_ production efficiency.

**Figure 2:**
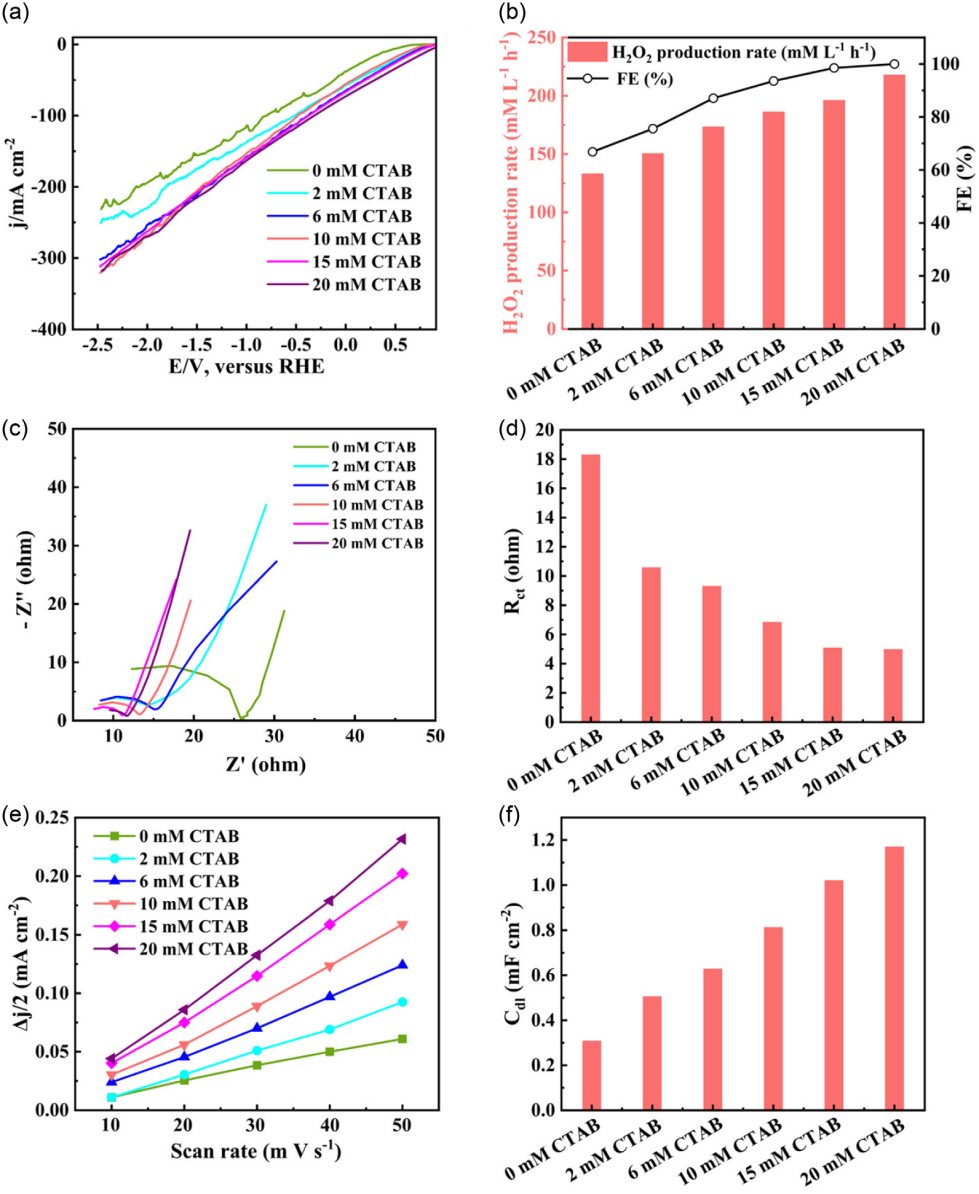
Promotion effect of CTAB on H_2_O_2_ production rate and reaction kinetic. a) LSV curve. b) H_2_O_2_ production rate and corresponding FE (%) as a function of CTAB concentration. c) Nyquist plots. d) Charge transfer resistance (*R*_ct_). e) Current density as a function of scan rate. f) Electric double‐layer capacitance (*C*_dl_) with various CTAB concentration.

The catalytic performance at various CTAB concentrations ranging from 2.0 to 20.0 mM was studied under a constant applied current density of 250.0 mA cm^−2^ (**Figure** **2**). As shown in Figure 2a, the 2e^−^ ORR current density increased across various CTAB concentrations. Correspondingly, the H_2_O_2_ production rate showed remarkable improvements with increasing CTAB concentrations, as shown in Figure 2b. The maximum H_2_O_2_ production rate of 217.8 mM L^−1^ h^−1^ with an impressive FE of ≈100.0% could be achieved at 20 mM CTAB, representing a 1.58‐fold improvement comparing with the H_2_O_2_ yield in the absence of CTAB.

To understand the underlying mechanistic insight into cationic surfactant‐modified interfacial microenvironment in promoting the H_2_O_2_ production rate, a series of electrochemical characterization and an in‐depth kinetic analyses were performed. Figure 2c shows the Nyquist plots for each electrolyte containing different concentrations of CTAB at 0.6 V_RHE_. The charge transfer resistance (*R*_ct_) could be determined by fitting the equivalent circuit model (Figure S5, Supporting Information), and it could be observed from Figure 2d that *R*_ct_ decreased significantly with increasing CTAB concentration, specifically, from 18.3 ohm (in the absence of CTAB) to 10.5 ohm in 2 mM CTAB and 4.9 ohm in 20 mM CTAB, which showed 3.7 times decreasing. The decrease of *R*_ct_ might be attributed to the accumulated charges at the electrode–electrolyte interface and the acceleration of the electron transfer process.^[^ ^33^ ^]^

Furthermore, the electrochemical electric double‐layer capacitance (*C*_dl_) could be determined by collecting cyclic voltammograms in a non‐Faradaic region at different scan rates (Figure 2e). Figure 2f reveals that the *C*_dl_ increased obviously with addition of CTAB surfactant, indicating the increase of electrochemical surface area and an improved capacitive behavior, which aligned well with the above observed decrease of *R*_ct_ and corresponding improvement of H_2_O_2_ production rate. This behavior should be ascribed to the dynamic adsorption of CTAB on the electrode that might lead to charge accumulation at the electrode–electrolyte interface within the electric double layer, resulting in efficient stabilization of HO_2_^−^ intermediates and inhibiting its further reaction or reduction.^[^ ^21^ ^]^

### 2.2 Mechanistic Understanding of Cationic Surfactant‐Modified Interfacial Microenvironment

**Figure 3:**
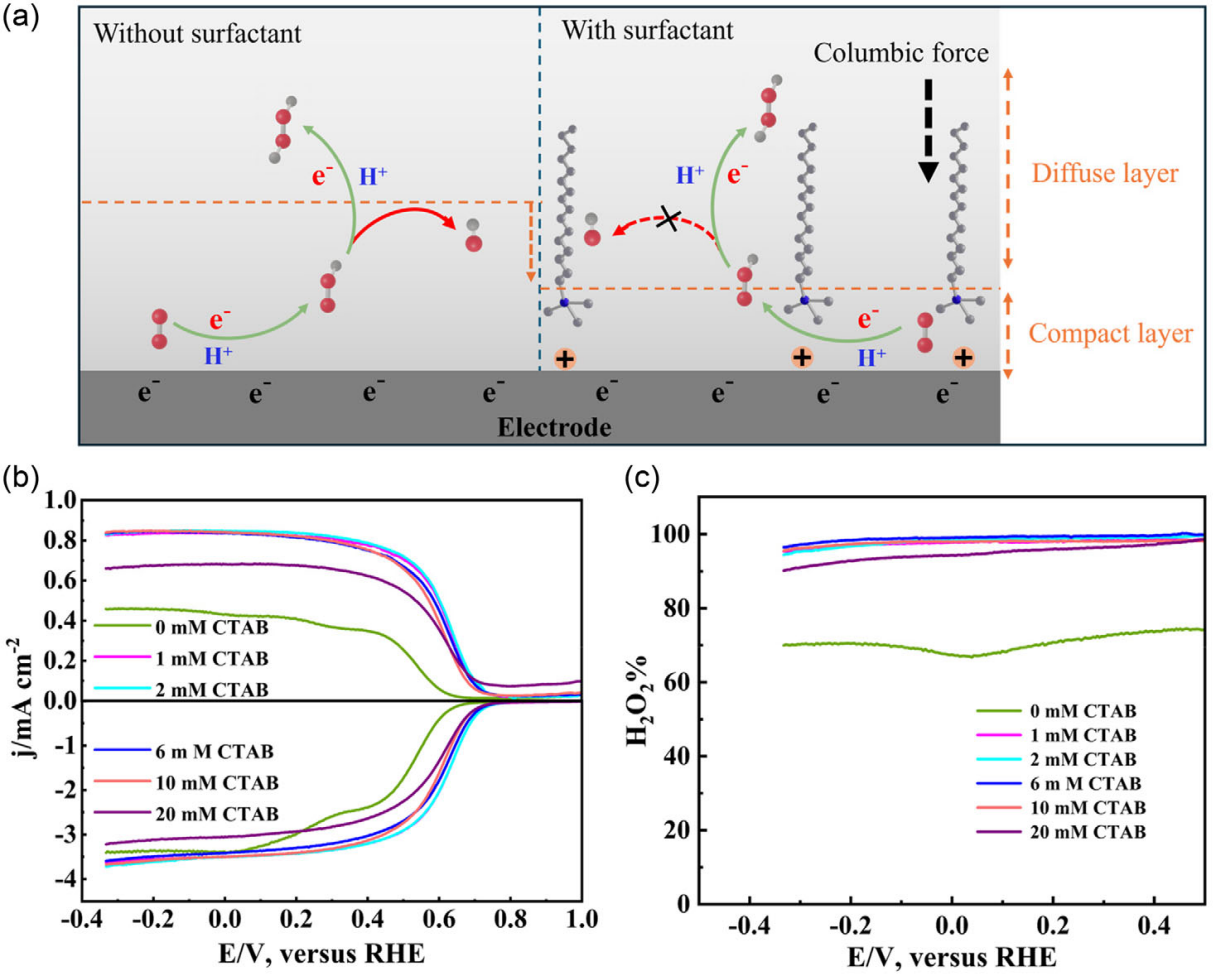
Reaction mechanism: a) simplified reaction mechanism with and without CTAB; b) SCV‐RRDE profiles; and c) hydrogen peroxide selectivity (H_2_O_2_%) of CB in various CTAB concentrations of 0 mM CTAB, 1 mM CTAB, 2 mM CTAB, 6 mM CTAB, 10 mM CTAB, and 20 mM CTAB.

Based on these above findings, we proposed that the enhanced H_2_O_2_ production rate results from the modulation of the electric double layer and the strengthening local electric field at the electrode–electrolyte interface (**Figure** **3**). The positively charged ammonium head group of CTAB adsorbs onto the negatively charged carbon electrode surface via columbic force, leading to charge accumulation at the electrode–electrolyte interface. The accumulated charge strengthens the local electric field, which is crucial in enhancing accelerating electron transfer kinetics, as evidenced by the significant reduction in charge transfer resistance (*R*_ct_) observed in electrochemical impedance spectroscopy measurements (Figure 2c,d). The reduction in *R*_ct_ suggests that CTAB promotes charge redistribution and lowers the reaction barrier for oxygen reduction, thereby effectively stabilizing HO_2_^−^ intermediates and suppressing their further reduction to H_2_O, making the 2e^−^ ORR pathway more favorable. Control experiment using RRDE (Figure 3b,c) proved well this hypothesis. As shown in Figure 3b, the ORR on CB follows a two‐step reduction in 0.1 M KOH (0 mM CTAB) in RRDE. The first reduction in the voltage range of 0.3–0.65 V_RHE_ should be corresponded to the reduction of O_2_ into HO_2_^−^, and the second step involved the subsequent reduction of HO_2_^−^ into OH^−^ at the voltage <0.35 V_RHE_. In sharp contrast, the second reduction step became negligible upon the addition of CTAB into the electrolyte, indicating the effective selective reduction of O_2_ to H_2_O_2_, evidenced by higher H_2_O_2_ selectivity over 95.0% (Figure 3c).

Additionally, the observed increase in electric double‐layer capacitance (*C*_dl_) (Figure 2f) indicates an enhancement in local charge density at the electrode interface. The higher charge density strengthens the electrostatic stabilization of the negatively charged HO_2_^−^ intermediates, preventing their desorption and further reduction as illustrated in Figure 3a. This effect aligns with the improved H_2_O_2_ selectivity observed in RRDE measurements, confirming that CTAB‐modified microenvironments promote the 2e^−^ ORR pathway while effectively suppressing competing reactions. These findings highlight the role of interfacial charge redistribution and electrostatic stabilization in governing H_2_O_2_ production efficiency, providing insights into the rational design of surfactant‐modified catalytic systems for selective electrosynthesis.

### 2.3 Optimization of the Carbon Catalyst

**Scheme 2:**
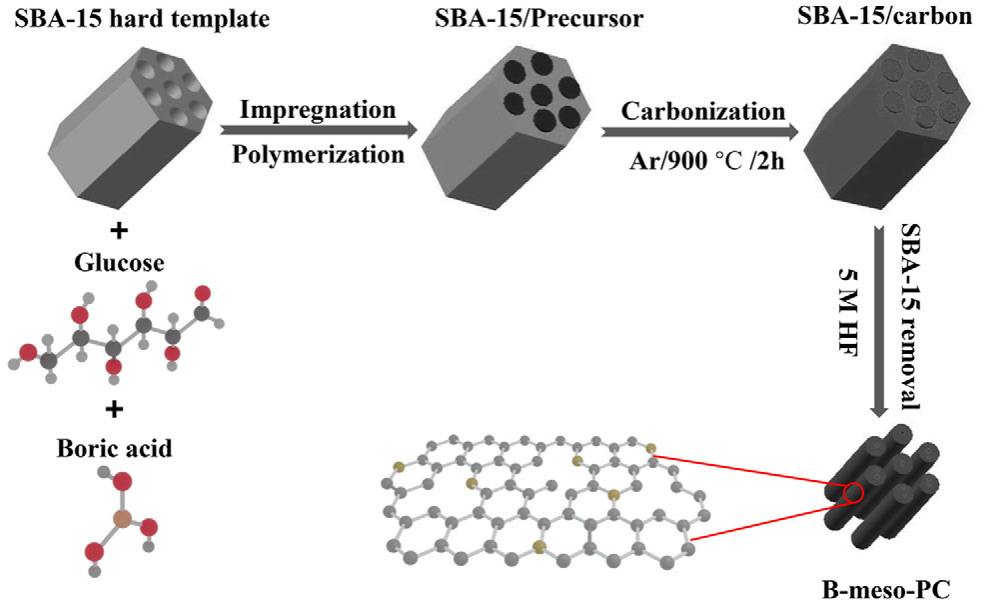
Schematic illustration of B‐meso‐PC synthesis process.

In addition to the interfacial microenvironment modulation, the catalyst material itself is another key factor that significantly impacts the overall efficiency and selectivity of the 2e^−^ ORR process. Our previous work had demonstrated that mesoporous carbon materials are superior catalysts for 2e^−^ ORR due to their ordered porous structure, large surface area, plenty of accessible active sites, and improved reactant diffusion.^[^ ^30^ ^]^ In addition, heteroatom doping may introduce extra active sites, further enhancing oxygen reduction to H_2_O_2_.^[^ ^26^ ^]^ Inspired by these above pioneered results, a novel B‐meso‐PC with enhanced surface area and optimized porous structure was synthesized in this section to substitute commercial CB for higher 2e^−^ ORR performance. The B‐meso‐PC was synthesized using SBA‐15 hard template method. The typical synthesis process involved impregnating glucose and boric acid into the pores of SBA‐15 hard templates, followed by subsequent polymerization, carbonization, and template removal procedures (**Scheme** **2**).

**Figure 4:**
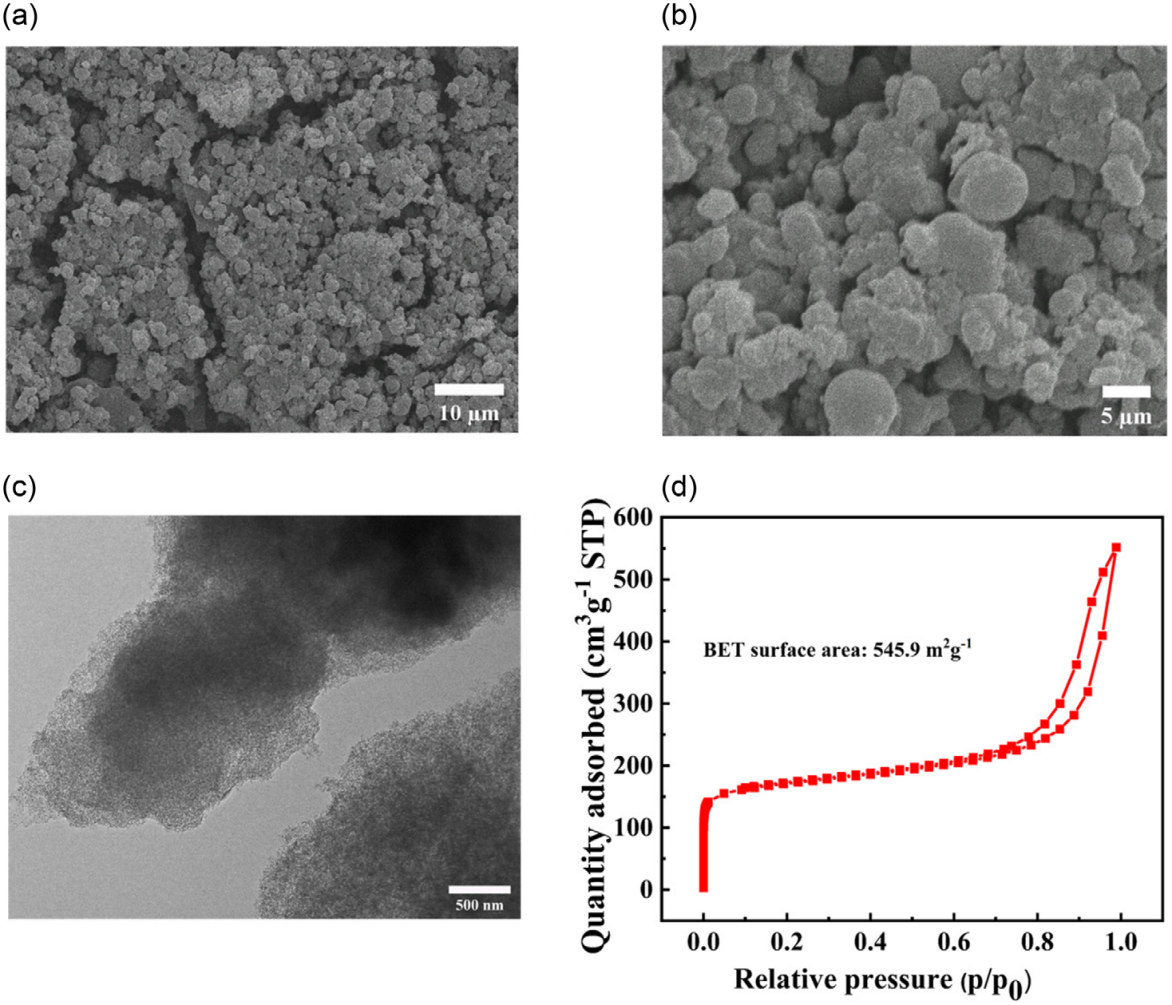
Material characterizations of catalysts: a,b) SEM image, c) TEM image, and d) N_2_‐adsorption–desorption isotherms of B‐meso‐PC.

As illustrated in **Figure** **4**a,b, the scanning electron microscope (SEM) images of B‐meso‐PC revealed well‐distributed pores across the entire surface, suggesting a highly porous structure of the synthesized B‐meso‐PC. The transmission electron microscope (TEM) image (Figure 4c) further confirmed the high density of well‐defined mesoporous structure within the synthesized B‐meso‐PC material. The mesoporous structure could not only enhance the surface area but also facilitated the efficient diffusion of reactants, further contributing to the improved catalytic performance in 2e^−^ ORR process.^[^ ^34^, ^35^ ^]^ Moreover, N_2_‐adsorption–desorption isotherm measurements, which is shown in the Figure 4d, exhibited a typical type‐IV isotherm, indicating a substantial volume of mesopores. B‐meso‐PC exhibits a notably high specific surface area of 545.9 m^2^ g^−1^, which is significantly higher than that of CB (59.0 m^2^ g^−1^, Table S2, Supporting Information).

**Figure 5:**
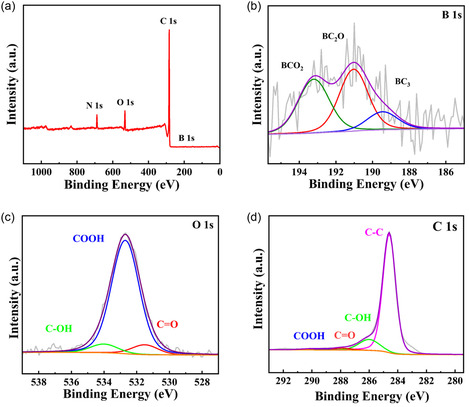
XPS characterizations: a) XPS survey, b) high‐resolution B 1*s* XPS spectra, c) high‐resolution O 1*s* XPS spectra, and d) high‐resolution C 1*s* XPS spectra of B‐meso‐PC.

The chemical composition of B‐meso‐PC was determined using XPS measurement (**Figure** **5**). As shown in Figure 5a, the XPS survey spectra indicated that B‐meso‐PC contains carbon, nitrogen, oxygen, and boron elements. The atomic percentages shown in Table S3, Supporting Information further verify the successful incorporation of boron into the carbon framework. The high‐resolution B 1*s* XPS spectra (Figure 5b) could be deconvoluted into three distinct peaks located at 190.0, 191.2, and 192.7 eV, corresponding to BC_3_, BC_2_O, and BCO_2_, respectively.^[^ ^36^ ^]^ The high‐resolution O 1*s* XPS spectra (Figure 5c) could be deconvoluted into three peaks at 531.4, 532.7, and 534.0 eV, corresponding to C=O, COOH, and C—OH surface functionalities, respectively. The high‐resolution C 1*s* XPS spectra (Figure 5d) exhibited four peaks at 284.6, 286.0, 287.7, and 289.4 eV, corresponding to the binding energy signals of C—C, C—OH, C=O, and COOH groups, respectively.

**Figure 6:**
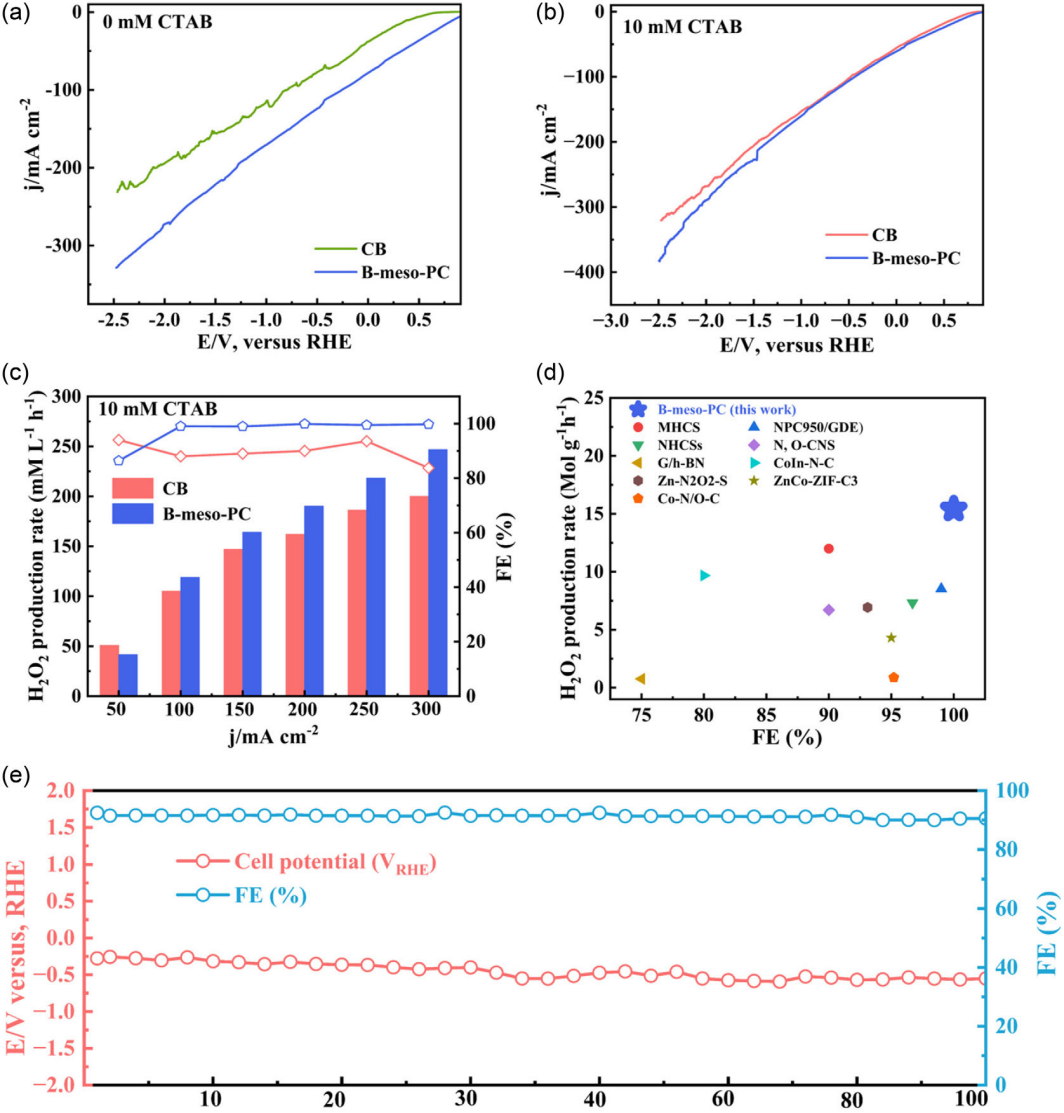
Comparison of H_2_O_2_ production performance of CB and B‐meso‐PC in flow‐cell reactor. a) LSV curve of CB and B‐meso‐PC in 0 mM CTAB, b) LSV curve of CB and B‐meso‐PC in 10 mM CTAB, c) H_2_O_2_ production rate and corresponding FE (%) of CB and B‐meso‐PC in 10 mM CTAB, d) comparison of H_2_O_2_ production rate and corresponding FE (%) of B‐meso‐PC with previously reported works, and e) long‐term stability test of B‐meso‐PC catalyst under 100 mA cm^−2^ in 2 mM CTAB.

The electrochemical performance of B‐meso‐PC was also evaluated in the electrolyte in the absence and presence of CTAB (10 mM CTAB), as shown in **Figure** **6**. The LSV of B‐meso‐PC exhibited much higher 2e^−^ ORR current density of 330.0 mA cm^−2^ than CB (227.0 mA cm^−2^) in the absence of CTAB (Figure 6a). The catalytic activity of B‐meso‐PC could be further improved in the presence of CTAB (10 mM CTAB), achieving a higher 2e^−^ ORR current density at 382.0 mA cm^−2^ (Figure 6b), which is much higher that recently reported cutting‐edge‐electrocatalyst material for H_2_O_2_ production (Table S4, Supporting Information). In addition, the B‐meso‐PC in CTAB system exhibited a relatively high H_2_O_2_ production rate of 246.7 mM L^−1^ h^−1^ with FE of 100.0% at a constant current density of 300.0 mA cm^−2^ (Figure 6c), representing a 1.80‐fold improvement comparing with conventional reaction system.

As shown in Figure 6d, the proposed B‐meso‐PC with CTAB system exhibited higher activity in electrochemical synthesis of H_2_O_2_ via 2e^−^ ORR comparing with other typical reaction systems from the viewpoint of both production rate and FE^[^ ^4^, ^37^, ^38^, ^39^, ^40^, ^41^, ^42^, ^43^, ^44^ ^]^ (Table S5, Supporting Information). In addition, the stability of the catalyst is also crucial for its potential industrial applications. As shown in Figure 6e, the proposed B‐meso‐PC with CTAB system demonstrated excellent catalytic stability, maintaining its high 2e^−^ ORR performance for 100 h at a fixed current density of 100.0 mA cm^−^^2^ in 2 mM CTAB with negligible voltage increment. Furthermore, the FE remains stable (>90%) during continuous operation, suggesting its unique advantages and great potential practical applications. These findings emphasized the importance for the synergistic adjustment of interfacial microenvironmental engineering and catalyst design strategies for boosting the catalytic activity of 2e^−^ ORR process.

## 3 Conclusion

In summary, we demonstrated here the positive impact of cationic surfactant‐modified interfacial microenvironment strategy on the catalytic performance of electrochemical synthesis of H_2_O_2_ via carbon materials in alkaline flow‐cell reactors. Notably, the introduction of CTAB into the electrolyte optimized the key performance metrics for 2e^−^ ORR process, including current density, H_2_O_2_ production rate, and reaction kinetics. Detailed activity and structure analysis revealed that CTAB modulated the electrode–electrolyte interface by strengthening the electric field within electric double layer, which was recognized as the physicochemical nature behind this enhancement effect. In addition, the performance of the reaction system could be further enhanced via substituting the commercial CB catalyst with well‐designed B‐doped mesoporous carbon material, which could further increase the H_2_O_2_ production rate, markedly outperforming other typical state‐in‐art reaction systems. This improvement should be attributed to the synergy between the modified interfacial microenvironment at the electrode–electrolyte interface and the unique tailored porous structure and boron heteroatom doping of carbon catalytic material. The present study represents a powerful strategy for designing efficient and scalable electrochemical system for potential industrial H_2_O_2_ production.

## 4 Experimental Section

### Chemicals and Reagents

Commercial CB (Ketjen black), glucose, sulfuric acid (H_2_SO_4_), isopropyl alcohol, and hydrofluoric acid (HF) were purchased from Sinopharm Chemical Co., Ltd. Boric acid and CTAB cationic surfactant were purchased from Alfa Aesar Chemicals Co., Ltd. Cerium sulfate (Ce(SO_4_)_2_) was purchased from Damas‐beta Chemicals Co., Ltd. Mesoporous silica template (SBA‐15) was purchased from Xfnano Material Tech Co., Ltd. Nafion solution (D520, 5 wt%) and proton exchange membrane (PEM, Nafion N117) were purchased from Sci Materials Hub.

### Synthesis of B‐meso‐PC

For synthesizing B‐meso‐PC, 1.25 g glucose was dissolved in 5.0 mL of purified water and stirred for ≈30.0 min. Subsequently, 1.0 g SBA‐15, 1.0 g boric acid, and 0.14 g H_2_SO_4_ were added to the glucose solution and stirred for 6.0 h. The resulting product was placed on a Teflon‐lined autoclave and kept at 100.0 °C for 6.0 h, followed by an additional 6.0 h at 160.0 °C. The resulted mixture was impregnated again with 5.0 mL H_2_O, 0.8 g glucose, and 0.14 g H_2_SO_4_, followed by heating at 100.0 °C for 6.0 h and 160.0 °C for 6 h. The obtained solid product was carbonized at 900.0 °C for 2.0 h under Ar flow. To remove the silica template, the SBA‐15/carbon composite was dispersed in 5.0 M HF (Sinopharm, China) at room temperature, and the mixture was stirred for 2 days. The resulting suspension underwent centrifugation, raised with water, and dried at 60.0 °C, to obtain the B‐meso‐PC.

### Catalyst Characterization

The morphological and microstructural properties of the catalyst materials were analyzed using SEM and a TEM (FEI Tecnai G2 F20). The surface chemical compositions were quantified by XPS (ESCALAB250). The porosity of catalyst materials and pore size distribution were analyzed using the Brunauer–Emmett–Teller technique.

### Flow‐Cell Assembly and Electrochemical Measurement

The electrochemical flow‐cell measurement was carried out using a three‐electrode flow‐cell configuration with two chambers (anolyte and catholyte) separated by a PEM (Nafion 117). The ORR catalyst was loaded onto a gas diffusion layer (carbon paper), and oxygen (O_2_) was fed through the backside of the gas diffusion layer via a titanium gas flow chamber. A commercial Ru–Ir–Ti mesh was employed as the anode catalyst for the oxygen evolution reaction. The catholyte (1.0 M KOH or 1.0 M KOH‐containing CTAB) and the anolyte (1.0 M KOH) were pumped at a constant flow rate of 5.0 mL min^−1^ using a peristaltic pump. The electrode area exposed to the electrolyte is 4.0 cm^2^ with mass loading of 0.4 mg cm^−2^. The catalyst ink was prepared by mixing 40.0 mg of catalyst powder with 4.0 mL of 2‐propanol, 1.0 mL of methanol, and 80.0 μL of 5.0% Nafion solution, followed by sonication for 30.0 min.

### Quantification of H_2_O_2_ Concentration

To determine the concentration of H_2_O_2_ produced in the electrochemical reaction, we employed the cerium sulfate (Ce(SO_4_)_2_) titration method, which is based on the redox reaction between Ce^4+^ and H_2_O_2_. In this reaction, Ce^4+^ (yellow) is reduced to Ce^3+^ (colorless) upon reacting with H_2_O_2_, and the resulting decrease in absorbance at 318.0 nm is directly proportional to the H_2_O_2_ concentration. The calibration curve was established by measuring the absorbance of Ce^4+^ solutions (0.1–1.0 mM) in 1.0 M H_2_SO_4_ at 318.0 nm using UV–vis spectroscopy (Figure S1a, Supporting Information). To quantify H_2_O_2_, 50 μL of the electrolyte solution containing H_2_O_2_ was mixed with a known volume of 1.0 mM (Ce(SO_4_)_2_). After the reaction, the absorbance of the remaining Ce^4+^ was recorded, and the decrease in absorbance was used to determine the H_2_O_2_ concentration using the calibration curve (Figure S1b, Supporting Information). Finally, the H_2_O_2_ concentration was calculated according to Equation (2), based on the redox reaction described in Equation (1).

(1) $$2{\text{Ce}}^{4+}+H_{2}O_{2}\to \;2{\text{Ce}}^{3+}+2H^{+}+O_{2}$$

(2) $$C\left(H_{2}O_{2}\right)=\frac{C_{0}\left({\text{Ce}}^{4+}\right)-C\left({\text{Ce}}^{4+}\right)\times V\left({\text{Ce}}^{4+}\right)}{2\times V\left(H_{2}O_{2}\right)}\;$$

where C_0_(Ce^4+^) is the initial concentration, C(Ce^4+^) is the final concentration, V(Ce^4+^) is the consumed volume, and V(H_2_O_2_) is the titrated volume.

The FE was calculated from Equation (3):

(3) $$\text{FE}\left(\%\right)=\frac{H_{2}O_{2}\times \text{flow rate}\times 2\times 96485}{I}\;\times 100\%$$

where the flow rate is electrolyte flow rate (mL s^−1^), 96 485 is Faradic constant (C mol^−1^) and H_2_O_2_ is H_2_O_2_ concentration (mol L^−1^), and *I* is current density (mA).

## Conflict of Interest

The authors declare no conflict of interest.

## Author Contributions

**Abdalazeez Ismail Mohamed Albashir**: methodology; writing and validation. **Yunlong Li**: investigation and software. **Jing Dou** and **Ke Qi**: methodology and investigation. **Wei Qi**: conceptualization; supervision; writing—review and editing.

## Supporting information

Supplementary Material

## Data Availability

The data that support the findings of this study are available from the corresponding author upon reasonable request.
