# Supplementary material for: Electrosynthesis of Hydrogen Peroxide at Industrial‐Level Current Density in Flow‐Cell System: Interfacial Microenvironment Regulation and Catalyst Design
Source: Small Sci. 2025 May 19;5(6):2500017. doi: 10.1002/smsc.202500017 (PMC12168599; doi:10.1002/smsc.202500017)
Supplement: Supplementary file 1 — Supplementary Material [file SMSC-5-2500017-s001.pdf]

Supporting information

Electrosynthesis of Hydrogen Peroxide at Industrial-level Current Density in Flow-cell System: Interfacial Microenvironment Regulation and Catalyst Design

Abdalazeez Ismail Mohamed Albashir [a, b]*, Yunlong Li* [a, b]*, Jing Dou* [a, b]*, Ke Qi* [a, b]*, Wei Qi* [a, b] *

[a] *School of Materials Science and Engineering, University of Science and Technology of China, Shenyang 110016, Liaoning, People's Republic of China*

[b] *Shenyang National Laboratory for Materials Science, Institute of Metal Research, Chinese Academy of Sciences, Shenyang, Liaoning, People's Republic of China*

*Corresponding author: wqi@imr.ac.cn

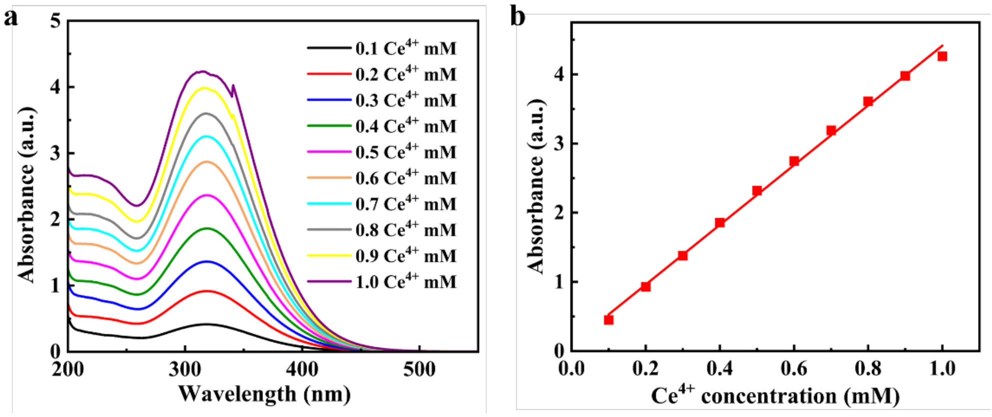

Fig. S1. UV-vis. (a) UV-vis absorbance of different concentrations of Ce^{4+}, and (b) Calibration curve of different concentrations of Ce^{4+}.

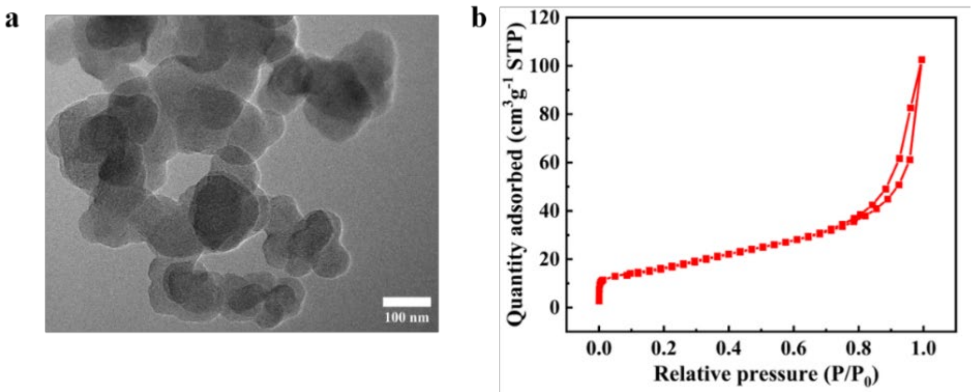

Fig. S2. Material characterizations of catalysts**.** (a) SEM image, and (b) N_2-adsorption-desorption isotherms of CB.

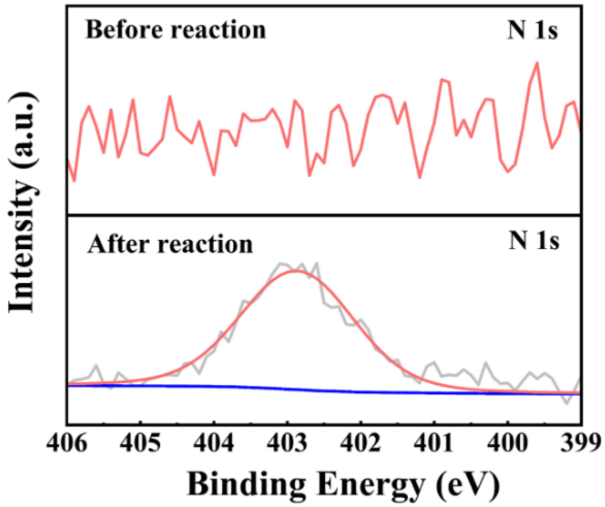

Fig. S3. XPS characterizations. N 1s XPS spectra before and after electrochemical reactions.

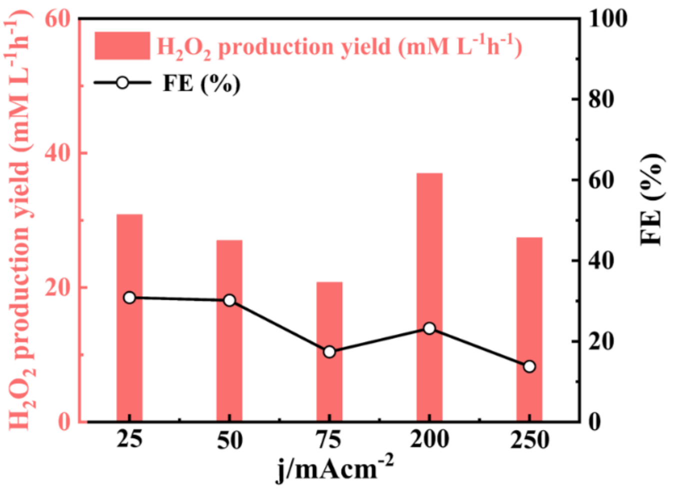

Fig. S4. H_2O_2 production performance in flow cell reactor. H_2O_2 production yield and corresponding FE (%) of CB in 1.0 M KOH containing 10 mM of sodium 1-hexadecanesulfonate anionic surfactant.

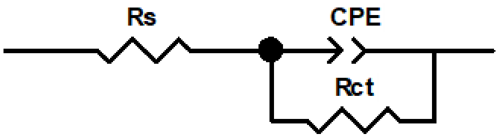

Fig. S5. The equivalent circuit diagrams. The equivalent circuit diagram in at 0.6 V_{RHE}.

Table S1. Chemical composition of CB before and after electrochemical reaction**.**

**Catalyst**	**Carbon (%)**	**Nitrogen (%)**	**Oxygen (%)**	Bromide **(%)**
Before reaction	99.49	0.21	0.30	0
After reaction	97.00	1.41	1.50	0.09

Table S2. Specific surface area and pore volume of CB and B-meso-PC.

**Catalyst**	**BET (m^2 g^{-1})**	**V_t (cm^3 g^{-1})**	**V_{micro} (cm^3 g^{-1})**
CB	59.05	0.158	0.144
B-meso-PC	545.9	0.853	0.631

Table S3. Chemical composition of CB and B-meso-PC.

**Catalyst**	**Carbon (%)**	**Nitrogen (%)**	**Oxygen (%)**	**Boron (%)**
CB	99.49	0.21	0.30	0
B-meso-PC	91.58	0.75	6.53	0.60

Table S4. Comparison of H_2O_2 production rate with previously reported electrocatalysts.

**Catalyst**	**j/mA cm^{-2}**	**Potential (V_{RHE})**	**Catalyst loading (mg)**	**Stability (h)**	**Ref**
**B-meso-PC**	382.0	-2.5 - 0.9	0.4	100	This work
**BN-C-1**	>100	-0.2 - 1.0	1	12	[1]
**MHCS**	>300	0.0 - 0.8	0.158	16	[2]
**NBO-G/CNTs**	>100	0.6 - 0.8	1.0	12	[3]
**CB**	200	-2.5 - 0.0	-	20	[4]
**N-C**	300	0.55	0.5	-	[5]
**B–C**	300	0.68	0.5	30	[6]
**BBL-PcNi**	>220	0.0 - 1.0	0.3	200	[7]
**Pb SA/OC**	205	0.0 - 0.8	1	100	[8]
**CoNCB**	>100	0.3 - 0.9	0.2	5	[9]

Table S5. Comparison of H_2O_2 production rate with previously reported electrocatalysts.

Catalyst	Electrolyte	H_2O_2 rate (Mol g^{-1} h^{-1})	FE (%)	Current density (mA cm^{-2})@potential	Ref.
B-meso-PC	1.0 M KOH +10 mM CTAB	15.42	100	300	This work
CB	1.0 M KOH +10 mM CTAB	13.56	83.75	300	This work
CB	1.0 M KOH	8.56	57.37	300	This work
Mesoporous carbon spheres	0.1 M PBS	12.64	95	0.1 V_{RHE}	[2]
Nitrogen-doped porous carbon nanopolyhedra	1.0 M KOH	8.53	95.0	100	[10]
Nitrogen doped hollow carbon nanospheres	0.1 M KOH	7.32	96.7	0.5 V_{RHE}	[11]
N, O co-doped carbon nanosheets	0.1 M K_2SO_4	6.705	90	0.2 V_{RHE}	[12]
graphene/hexagonal boron nitride	0.1 M KOH	0.762	75	0.2 V_{RHE}	[1]
CoIn-N-C	0.1 M $HClO_4$	9.68	90	100	[13]
Zn-N_2O_2-S	0.1 M KOH	6.924	93.1	80	[14]
ZnCo-ZIF-C3	0.1 M PBS	4.35	75	60	[15]
Co-N/O-C	0.1 M KOH	0.88	95.2	100	[16]

Reference

[1] M. Fan, Z. Wang, Y. Zhao, Q. Yuan, J. Cui, J. Raj, K. Sun, A. Wang, J. Wu, H. Sun, B. Li, L. Wang J. Jiang, Porous heterostructure of graphene/hexagonal boron nitride as an efficient electrocatalyst for hydrogen peroxide generation, Carbon Energy **2023,** 5, 1–14.

[2] Q. Tian, L. Jing, H. Du, Y. Yin, X. Cheng, J. Xu, J. Chen, Z. Liu, J. Wan, J. Liu, J. Yang, Mesoporous carbon spheres with programmable interiors as efficient nanoreactors for H_2O_2 electrosynthesis, Nat. Commun. **2024,** 15, 1–14.

[3] M. Fan, Z. Wang, K. Sun, A. Wang, Y. Zhao, Q. Yuan, R.b. Wang, J.t Raj, J.g. Wu, J.c Jiang, L. Wang, N-B-OH Site-Activated Graphene Quantum Dots for Boosting Electrochemical Hydrogen Peroxide Production, Adv. Mater. **2023**, 35, 2209086.

[4] Z. Adler, X. Zhang, G.x Feng, Y.p. Shi, P. Zhu, Y. Xia, X.n. Shan, and H.ti Wang , Hydrogen Peroxide Electrosynthesis in a Strong Acidic Environment Using Cationic Surfactants, Precis. Chem. **2024**, 2, 129−137.

[5] S. Rawah, M. Albloushi, W. Li, Electro-synthesis of pure aqueous H_2O_2 on nitrogen-doped carbon in a solid electrolyte flow cell without using anion exchange membrane, Chem. Eng. J. **2023**, 466, 143282.

[6] Y. Xia, X. Zhao, C. Xia, Z. Wu, .P Zhu, J. Kim, X. Bai, G. Gao, Y. Hu, J. Zhong, Y. Liu, H. Wang, Highly active and selective oxygen reduction to H_2O_2 on boron-doped carbon for high production rates, Nat. Commun. **2021**, **12**, 4225.

[7] M. Di Zhang, J. R. Huang, C. P. Liang, X. M. Chen, and P. Q. Liao, Continuous Electrosynthesis of Pure H_2O_2 Solution with Medical-Grade Concentration by a Conductive Ni-Phthalocyanine-Based Covalent Organic Framework, J. Am. Chem. Soc. **2024***,* 146, 31034−31041.

[8] X. Zhou, Y. Min, C.m Zhao, C. Chen, M-KunKe, S-L. Xu, J-J. Chen,Y. Wu, H.QingYu Constructing sulfur and oxygen super-coordinated main-group electrocatalysts for selective and cumulative H2O2 production, Nat. Commun. **2024**, 15, 193.

[9] L Liu, L. Kang, J. Feng, D. G. Hopkinson, C. S. Allen,Y. Tan, H. Gu, I. Mikulska, V. Celorrio, D. Gianolio, T. Wang, L. Zhang, K. Li, J. Zhang, J. Zhu,G. Held, P. Ferrer, D. Grinter, J. Callison, M. Wilding, S. Chen, I. Parkin, G. He, Atomically dispersed asymmetric cobalt electrocatalyst for efficient hydrogen peroxide production in neutral media, Nat

Commun **2024**, 15, 4079.

[10] P. Cao, X. Quan, K. Zhao, X. Zhao, S. Chen, and H. Yu, Durable and Selective Electrochemical H_2O_2 Synthesis under a Large Current Enabled by the Cathode with Highly Hydrophobic Three-Phase Architecture, *A* ACS Catal. **2021,** 11, 13797–13808.

[11] Z. Xu, Z. Ma, K. Dong, J. Liang, L. Zhang, Y. Luo, Q. Liu, J. You, Z. Feng, D. Ma, Y. Wang, X. Sun, Electrocatalytic two-electron oxygen reduction over nitrogen doped hollow carbon nanospheres," *Chem. Commun.* Chem. Commun. **2022,** 2, 5025–5028.

[12] L. Jing, Q. Tian, W. Wang, X. Li, Q. Hu, H. Yang, C. He, Unveiling Favorable Microenvironment on Porous Doped Carbon Nanosheets for Superior H2O2 Electrosynthesis in Neutral Media, Adv. Energy Mater. **2024**, 14, 2304418.

[13] J. Du, G. Han, W. Zhang, L. Li, Y. Yan, Y. Shi, X. Zhang, L. Geng, .Z Wang, Y. Xiong, G. Yin, C. Du, CoIn dual-atom catalyst for hydrogen peroxide production via oxygen reduction reaction in acid, Nat. Commun. **2023,** 14, 4766.

[14] G. Wei, Y. Li, X. Liu, J. Huang, M. Liu, D. Luan, S. Gao, X. Lou, Single-Atom Zinc Sites with Synergetic Multiple Coordination Shells for Electrochemical H_2O_2 Production, Angew. Chem. Int. Ed. **2023**, 62, e202313914.

[15] C. Zhang, L. Yuan, C. Liu, Z. Li, Y. Zou, X. Zhang, Y. Zhang, Z. Zhang, G. Wei, C. Yu, Crystal Engineering Enables Cobalt-Based Metal-Organic Frameworks as High-Performance Electrocatalysts for H_2O_2 Production, J. Am. Chem. Soc. **2023,** 145, 7791–7799.

[16] B. Li, M. Lan, L. Liu, D. Wang, S. Yang, Y. Sun, F. Xiao, J. Xiao, Continuous On-Site H_2O_2 Electrosynthesis via Two-Electron Oxygen Reduction Enabled by an Oxygen-Doped Single-Cobalt Atom Catalyst with Nitrogen Coordination, ACS Appl. Mater. Interfaces **2023**, 15, 37619–37628.
